# *CUX1*-related neurodevelopmental disorder: deep insights into phenotype-genotype spectrum and underlying pathology

**DOI:** 10.1038/s41431-023-01445-2

**Published:** 2023-08-30

**Authors:** Henry Oppermann, Elia Marcos-Grañeda, Linnea A. Weiss, Christina A. Gurnett, Anne Marie Jelsig, Susanne H. Vineke, Bertrand Isidor, Sandra Mercier, Kari Magnussen, Pia Zacher, Mona Hashim, Alistair T. Pagnamenta, Simone Race, Siddharth Srivastava, Zoë Frazier, Robert Maiwald, Matthias Pergande, Donatella Milani, Martina Rinelli, Jonathan Levy, Ilona Krey, Paolo Fontana, Fortunato Lonardo, Stephanie Riley, Jasmine Kretzer, Julia Rankin, Linda M. Reis, Elena V. Semina, Miriam S. Reuter, Stephen W. Scherer, Maria Iascone, Denisa Weis, Christina R. Fagerberg, Charlotte Brasch-Andersen, Lars Kjaersgaard Hansen, Alma Kuechler, Nathan Noble, Alice Gardham, Jessica Tenney, Geetanjali Rathore, Stefanie Beck-Woedl, Tobias B. Haack, Despoina C. Pavlidou, Isis Atallah, Julia Vodopiutz, Andreas R. Janecke, Tzung-Chien Hsieh, Hellen Lesmann, Hannah Klinkhammer, Peter M. Krawitz, Johannes R. Lemke, Rami Abou Jamra, Marta Nieto, Zeynep Tümer, Konrad Platzer

**Affiliations:** 1 Institute of Human Genetics, University of Leipzig Medical Center, Leipzig, Germany; 2 Department of Cellular and Molecular Biology, Centro Nacional de Biotecnología, Consejo Superior de Investigaciones Científicas (CNB-CSIC), Campus de Cantoblanco, Madrid, Spain; 3 Department of Neurology, Washington University in St Louis, St Louis, MO USA; 4 Dpt. of Clinical Genetics, Copenhagen University Hospital-Rigshospitalet, Copenhagen, Denmark; 5 Service de Génétique Médicale, CHU de Nantes, Nantes, France; 6 L’institut du thorax, Inserm, Cnrs, Univ Nantes, Nantes, France; 7 Randall Children’s Hospital at Legacy Emanuel, Portland, OR USA; 8 Epilepsy Center Kleinwachau, Radeberg, Germany; 9 NIHR Oxford Biomedical Research Centre, Wellcome Centre for Human Genetics, University of Oxford, Oxford, UK; 10 BC Children’s Hospital, University of British Columbia, Vancouver, BC Canada; 11 Department of Neurology, Boston Children’s Hospital, Boston, MA USA; 12 MVZ for Coagulation Diagnostics and Medical Genetics Cologne, ÜBAG Zotz/Klimas, Cologne, Germany; 13 MVZ Düsseldorf Zentrum, ÜBAG Zotz/Klimas, Düsseldorf, Germany; 14 Fondazione IRCCS Ca’Granda Ospedale Maggiore Policlinico, Milan, Italy; 15 Laboratory of Medical Genetics, Bambino Gesù Children’s Hospital, IRCCS, Rome, Italy; 16 Departmental Unit of Molecular and Genomic Diagnostics, Fondazione Policlinico Universitario A. Gemelli IRCCS, Rome, Italy; 17 Genetics Department, CHU Robert-Debré, AP-HP, Paris, France; 18 Medical Genetics Unit, A.O.R.N. San Pio, Benevento, Italy; 19 Department of Pediatrics, University of Maryland School of Medicine, Baltimore, MD USA; 20 Department of Clinical Genetics, Royal Devon University Healthcare NHS Trust, Exeter, UK; 21 Department of Pediatrics and Children’s Research Institute, Medical College of Wisconsin and Children’s Hospital of Wisconsin, Milwaukee, WI USA; 22 The Centre for Applied Genomics, The Hospital for Sick Children, Toronto, ON Canada; 23 Department of Molecular Genetics, University of Toronto, Toronto, ON Canada; 24 Laboratory of Medical Genetics, ASST Papa Giovanni XXIII, Bergamo, Italy; 25 Department of Medical Genetics, Kepler University Hospital Med Campus IV, Johannes Kepler University, Linz, Austria; 26 Department of Clinical Genetics, Odense University Hospital, Odense, Denmark; 27 HC Andersen Childrens Hospital, Odense University Hospital, Odense, Denmark; 28 Institut für Humangenetik, Universitätsklinikum Essen, Universität Duisburg-Essen, Essen, Germany; 29 Blank Children’s Developmental Center, Unity Point Health, Des Moines, IA USA; 30 North West Thames Regional Genetic Service, North West London Hospitals, London, UK; 31 Division of Medical Genetics, University of California, San Francisco, CA USA; 32 Dvision of Pediatric Neurology, University of Nebraska Medical Center, Omaha, NE USA; 33 Institute of Medical Genetics and Applied Genomics, University of Tübingen, Tübingen, Germany; 34 Division of Genetic Medicine, Lausanne Universitary Hospital and University of Lausanne, Lausanne, Switzerland; 35 Department of Pediatrics and Adolescent Medicine, Division of Pediatric Pulmonology, Allergology and Endocrinology, Comprehensive Center for Pediatrics, Medical University of Vienna, Vienna, Austria; 36 Vienna Bone and Growth Center, Vienna, Austria; 37 Department of Pediatrics, Medical University of Innsbruck, Innsbruck, Austria; 38 Institute of Human Genetics, Medical University of Innsbruck, Innsbruck, Austria; 39 Institute for Genomic Statistics and Bioinformatics, University Hospital Bonn, Rheinische Friedrich-Wilhelms-Universität Bonn, Bonn, Germany; 40 Institut für Humangenetik, Universitätsklinikum Bonn, Universität Bonn, Bonn, Germany; 41 Institute for Medical Biometry, Informatics and Epidemiology, University Hospital Bonn, Rheinische Friedrich-Wilhelms-Universität Bonn, Bonn, Germany; 42 Center for Rare Diseases, University of Leipzig Medical Center, Leipzig, Germany; 43 Kennedy Center, Department of Clinical Genetics, Copenhagen University Hospital-Rigshospitalet, Copenhagen, Denmark; 44 Department of Clinical Medicin, Faculty of Health and Medical Sciences, University of Copenhagen, Copenhagen, Denmark

**Keywords:** Neurodevelopmental disorders, Epilepsy

## Abstract

Heterozygous, pathogenic *CUX1* variants are associated with global developmental delay or intellectual disability. This study delineates the clinical presentation in an extended cohort and investigates the molecular mechanism underlying the disorder in a *Cux1*^+/−^ mouse model. Through international collaboration, we assembled the phenotypic and molecular information for 34 individuals (23 unpublished individuals). We analyze brain CUX1 expression and susceptibility to epilepsy in *Cux1*^*+/−*^ mice. We describe 34 individuals, from which 30 were unrelated, with 26 different null and four missense variants. The leading symptoms were mild to moderate delayed speech and motor development and borderline to moderate intellectual disability. Additional symptoms were muscular hypotonia, seizures, joint laxity, and abnormalities of the forehead. In *Cux1*^*+/*−^ mice, we found delayed growth, histologically normal brains, and increased susceptibility to seizures. In *Cux1*^*+/*−^ brains, the expression of *Cux1* transcripts was half of WT animals. Expression of CUX1 proteins was reduced, although in early postnatal animals significantly more than in adults. In summary, disease-causing *CUX1* variants result in a non-syndromic phenotype of developmental delay and intellectual disability. In some individuals, this phenotype ameliorates with age, resulting in a clinical catch-up and normal IQ in adulthood. The post-transcriptional balance of CUX1 expression in the heterozygous brain at late developmental stages appears important for this favorable clinical course.

## Introduction

*CUX1* is a conserved mammalian homolog of *Drosophila melanogaster* Cut that encodes for two types of proteins through alternative splicing: cut-like homeobox 1 (CUX1) and cut alternately spliced protein (CASP) [1]. CUX1 isoforms are transcription factors, whereas CASP are Golgi proteins that share with CUX1 the N-terminal region but not the DNA binding motifs [2]. CUX1 proteins comprise long and short isoforms. The full-length p200 isoform contains four DNA binding motifs, three CUT repeats, and a homeodomain. It is proteolytically cleavaged into two shorter isoforms during the cell cycle. Additionally, alternative splicing generates several other shorter CUX1 isoforms [1]. p200 CUX1 acts as a transcriptional repressor, whereas short CUX1 isoforms can activate or suppress transcription [1, 3].

In mammals, many tissues express *CUX1/Cux1*, including the developing and mature central nervous system [4]. In the human brain, most pyramidal neurons in layers (L) 2–5 of the cortex and some subpopulations of hippocampal neurons express *CUX1* [5, 6]. *CUX1* active enhancers contain human accelerated regions. These are regions conserved across most mammals but highly divergent in humans that might have contributed to acquiring human traits such as cognition during evolution [7]. In agreement, variants in the *CUX1*-HAR region link to autism spectrum disorder (ASD) [7, 8]. In mice, CUX1 expression also defines mature and young pyramidal neurons of L2/3 and L4 (known as the upper layers), regulating the formation of dendritic arbors and callosal connections during development [9–11].

While all of the above support crucial roles of *CUX1/Cux1* in human neurodevelopment, these are only partially understood. Most known *CUX1* functions derive from studies on cancer cells, where the gene can act as an oncogene or a haploinsufficient tumor suppressor gene [12]. Studies in mice have struggled with the lethality of *Cux1* null variants. Homozygous knockout mutants (*Cux1*^−^^*/*−^) died shortly after birth due to underdeveloped lungs and respiratory failure [13], and only in outbred genetic backgrounds some mice survived to adulthood. These animals showed delayed growth and abnormal hair but no gross neurological symptoms [8]. *Cux1* heterozygous (*Cux1*^*+/*^^−^) animals are fertile and display no obvious phenotypes, but an in-depth analysis of the consequence of heterozygosis has never been pursued [13].

We previously reported nine individuals with heterozygous truncating variants in *CUX1*. All individuals had speech delay and most individuals exhibited motor delay and intellectual disability (ID). Furthermore, some individuals seemed to catch-up on their speech and motor development [14]. Notably, this study did not address the mechanisms underlying the haploinsufficiency of CUX1 in neurodevelopment.

Here, we review 11 previously [14–16] reported and 23 unpublished individuals with rare heterozygous *CUX1* variants, refining the associated clinical phenotype. Furthermore, we investigated *Cux1* expression and the phenotype of *Cux1*^*+/−*^ mice pertinent to the clinical phenotype of the individuals.

## Material and methods

### Study subjects

Through matchmaking and collaborative efforts [17, 18], we identified 23 previously unreported individuals with *CUX1* variants. Phenotype and genotype information was obtained from the referring physicians via a standardized questionnaire, together with brain MRI images. The description of the severity of the developmental delay and ID is based on IQ tests (based on the experience of each center, some tests are suitable for individuals younger than 6 years of age) and, if these were not available or not possible, on an age-appropriate assessment by the attending physician. Variants were identified using trio/single exome or genome sequencing, or chromosome microarray. When needed, we performed a segregation analysis using Sanger sequencing. We also included the information of eleven previously published individuals harboring heterozygous truncating or de novo missense *CUX1* variants [14–16]. For individual 25 we have collected further information. If not described otherwise, frequencies of clinical symptoms are described as the number of affected vs. assessed individuals. Variants were mapped to *CUX1* (NM_001202543.2) and *CASP* (NM_001913.4) GRCh37. We used the in silico scores CADD [19], REVEL [20] an MutPred2 [21] to predict the impact of the missense variants and gnomAD [22] to analyze the allel frequencies of variants in the general population. All variants are classified according the ACMG [23] guidelines.

### Facial analysis

We performed the GestaltMatcher approach [24] on CUX1 individuals to analyze the facial similarities among the nine individuals (Ind. 6, 7, 8, 9, 14, 18, 19, 25, and 28) who consented to the facial analysis (see Supplementary Materials for details).

### Animals

We obtained *Cux1*^*+/*^^−^ (*Cutl1*^*tm2Ejn*^) mice from A.J. van Wijnen (University of Massachusetts Medical School, Worcester, MA)[13] and maintained in a C57BL6JRccHsd background (Envigo Laboratories, formerly Harlan. Indianapolis, USA). We used WT, *Cux1*^*+/*^^−^, and *Cux1*^−^^*/*−^ littermates in all experiments. The day of appearance of a vaginal plug was defined as E0 and the day of birth, postnatal day 0 (P0). Animals were housed and maintained following the European Union Council Directive (86/609/European Economic Community).

### Western blotting

We analyzed the Cux1 p200 isoform expression in somatosensory cortex of WT, *Cux1*^*+/*^^−^ and *Cux1*^−^^*/*−^ mice via Western blot by using antibodies against CUX1 (Proteintech, 11733-1-AP; see Supplementary Materials for details).

### RT-qPCR analysis

Tissue dissection was performed as described above. cDNA synthesis and qPCR reactions were performed as previously described [11, 25] (see Supplementary Materials for details).

### Immunohistochemistry and Nissl staining

Mice were anesthetized using an intraperitoneal (i.p.) injection of ketamine and xylazine and perfused with formalin (Sigma). Brains were postfixed in formalin, cryoprotected in 30% sucrose, and cut in 50 µm free-floating cryosections. Sections were incubated with rabbit anti-Cux1 antibody (Santa Cruz Biotechnology, M222X), goat anti-rabbit Alexa 488 (Thermo Fisher Scientific, #A11034), and 4',6-diamidino-2-phenylindole dihydrochloride (DAPI) (Merck, #D9542). Nissl staining was performed as previously described [26].

### Confocal imaging, microscopy, and quantification

Images were taken using a Leica DM4B (Leica) with a 2.5X objective and LAS AF v1.8 software (Leica; see Supplementary Materials for details).

### Kainic acid model of epilepsy

10–13 week old mice were intraperitoneal injected with kainate (30 mg/kg of body weight) (Milestone PharmTech USA), and they were monitored for 120 min by video recording. We used a modified version of the Racine scale to score behavioral alterations [27]: 1-immobility, staring; 2-rigidity and automatisms; 3-unilateral forelimb clonus, forepaws on the belly; 4-rearing; 5-continuous rearing and falling; 6-total body clonus. Stages 1 and 2 were considered non-convulsive seizures, while stages 3–6 represented convulsive seizures. The maximum score of each animal and the latency to onset of stage 3 were quantified.

### Statistical analysis

Each experimental condition includes a minimum of three (RT-qPCR, immunostainings, and western blot) or ten (seizure induction) biological replicates. Results are expressed as the mean ± standard error of the mean (SEM) and compared using student *t* test, Mann-Whitney test, or ANOVA with posthoc comparison Sidak’s and Tukey’s test, as indicated in the corresponding figures, using GraphPad Prism v8 software.

## Results

In this study, we include 23 novel and 11 previously published individuals [14–16], from which 30 were unrelated, diagnosed with heterozygous variants in *CUX1*. The cohort compromises of nine females and 25 males with a median age of 7 years (ranging from 7 months to 78 years). Table 1 summarises  the clinical symptoms and Table S1 provides detailed clinical descriptions.

**Table 1:** Summary of clinical features of *CUX1*-related neurodevelopmental disorder.

Phenotype	HPO	missense		null variant		total	
		*n* = 4	in %	*n* = 29	in %	*n* = 34	in %
Intellectual and social development							
Intellectual disability/Developmental Delay		3/3	100%	30/30	100%	33/33	100%
Delayed speech and language development	HP:0000750	3/3	100%	28/29	97%	31/32	97%
unremarkable speech at last exam		0/3	0%	10/24	42%	10/27	37%
motor delay	HP:0001270	1/2	50%	23/30	77%	24/32	75%
no persistent motor problems after motor delay		0/1	0%	10/22	45%	10/23	43%
Intellectual disability	HP:0001249	2/2	100%	19/25	76%	21/27	78%
Intellectual disability, borderline	HP:0006889	0/2	0%	4/25	16%	4/27	15%
Intellectual disability, mild	HP:0001256	0/2	0%	8/25	32%	8/27	30%
Intellectual disability, moderate	HP:0002342	0/2	0%	7/25	28%	7/27	26%
Intellectual disability, severe	HP:0010864	2/2	100%	0/25	0%	2/27	7%
Autism spectrum disorder	HP:0000729	2/2	100%	4/24	17%	6/26	23%
Neurological and central nervous system							
Hypotonia	HP:0001252	1/2	50%	12/29	41%	13/31	42%
Seizure	HP:0001250	3/3	100%	5/29	18%	8/32	25%
Abnormality of movement	HP:0100022	1/2	50%	6/21	29%	7/23	30%
MRI abnormalities	HP:0012443	2/3	67%	10/17	59%	12/20	60%
Phenotypical abnormalities of body and face							
Broad forehead	HP:0000337	1/3	33%	7/23	30%	8/26	31%
Frontal bossing	HP:0002007	1/3	33%	3/23	13%	4/26	15%
Retrognathia	HP:0000278	0/3	0%	3/18	17%	3/21	14%
Macrocephaly	HP:0000256	0/3	0%	4/23	17%	4/26	15%
Low-set ears	HP:0000369	2/3	67%	5/22	23%	7/25	28%
Abnormality of the cardiovascular system	HP:0001626	1/3	33%	10/23	43%	11/26	42%
Joint laxity	HP:0001388	1/3	33%	7/17	41%	8/20	40%
Short stature	HP:0004322	0/3	0%	6/25	24%	6/28	21%
Abnormality of the male genitalia	HP:0010461	1/3	33%	9/13	69%	10/16	63%

### Phenotypic spectrum of the affected individuals—Intellectual and social development

All but one individual presented with delayed speech development (31/32, 97%). Individuals 4 and 21 initially presented with a disorder of motor development. Amongst individuals 6 years or older, formal IQ testing was available in ten Individuals. Suitable tests were provided for two individuals. If no formal test was available, the attending physician assessed the severity in an age-appropriate manner. ID was stratified as borderline (4/27, 15%), mild (8/27, 30%), moderate (7/27, 26%) and severe (2/27, 7%). Six individuals had an IQ in the normal range (6/27, 22%). Most individuals showed delayed motor development (24/32, 75%). The median age for walking was 20 months, and the medianfor first speech was 22.5 months. Six individuals were diagnosed with ASD (6/26, 23%).

We had previously noted that three individuals with a *CUX1* null variant caught up on developmental milestones [14]. Now, including the individuals presented in this study, developmental catch-up was observed in seven individuals (7/18, 39% from which this information was assessable; total 21%). Specifically, individual 6 presented speech and motor delay with first words occurring within 12–42 months and walking at 36 months of life. When he enrolled in a mainstream school, speech was comparable to children of the same age (developmental catch-up between 3 and 5 years of age). He received support throughout his school years in concordance with his lower IQ. At the age of 21 years, he had normal speech, borderline ID and was diagnosed with ASD. At age 36 months, individual 25 presented with significantly delayed speech (spoke 5–10 words) and motor development, muscular hypotonia, and macrocephaly. He could speak in 2–3 word sentences at 42 months, with further speech improvements at 4.5 years of age. At 5 years of age, the speech was comparable to children of the same age. However, due to muscular hypotonia motor development was still markedly delayed. At the age of seven, the individual enrolled in a mainstream school, albeit with integration aids for motor difficulties. Formal testing revealed an IQ of 90. At age nine, he was able to participate fully in school sports. The other six individuals presented variable delay of speech development in their earlier examinations, reporting first words within 12–42 months of age. However, they had normal speech or IQ at their last exams, performed at ages ranging from 8 to 55 years. These individuals were cognitively less affected (five individuals with normal IQ and three with borderline cognition) compared to individuals without catch-up development (two individuals with normal IQ, one with borderline, eight with mild, seven with moderate, and two with severe intellectual disability).

### Abnormalities of the nervous system

Muscular hypotonia was the most common neurological finding in the present cohort (13/31, 42%; median age of affected individuals: 5.3 years, ranged from 2 to 19 years). Seven individuals had mild cerebellar symptoms, including ataxia (7/23, 30%; median age of affected individuals: 6.6 years, ranged from 0.6 to 78 years), and eight individuals (8/32, 25%) developed seizures. The mean onset of seizures was 3 years and ranged between 1 and 6 years of age, with variable seizure types (including tonic-clonic and myoclonic seizures). All but two individuals became seizure-free (between 17 months and 22 years of age). Unfortunately, no further details regarding epilepsy type or EEG are available for the affected individuals. We could not observe a correlation between seizures and the severity of ID. Eight of the 20 individuals with available brain MRI imaging (performed and interpreted by each center) had no abnormalities. In the other 12 individuals, non-recurrent changes, such as a slightly prominent fourth ventricle, Chiari malformation, and white matter T2 hyperintensities, were observed (12/20, 60%; total 35%).

### Additional symptoms

Six individuals exhibited short stature (6/28, 21%), and eight showed joint laxity (8/20, 40%; total 23%). Abnormalities of the cardiovascular system, including persistent ductus arteriosus, atrial septal defect, and ventricular septal defect, were observed in eleven individuals (11/26, 42%; total 32%). Additional findings include mild scoliosis (3/21, 14%; total 9%) and genital malformations such as hypospadia, micropenis, and bilateral testicular ectopia in ten males (10/16, 63%; total 40%). The examined individuals had no apparent shared facial gestalt (Fig. S1). However, ten individuals displayed abnormalities in calvarial morphology, including macrocephaly, brachycephaly, plagiocephaly, and dolichocephaly. Eight individuals had a broad forehead (8/26, 31%), and four displayed frontal bossing (4/26, 15%). Seven individuals had low-set ears (7/25, 28%), and three had retrognathia (3/21, 14%). The physican’s examination was also consistent with the facial analysis performed by GestaltMatcher [24]. Although only nine individuals consented for a facial analysis, they might share a similar facial phenotype on the cohort level, as 78% of the distribution is below the threshold (Fig. S2). In the pairwise analysis (Fig. S3), it was clear that individuals 8, 9, 14, 18, and 19 formed one cluster, and individuals 6, 7, 25, and 28 were not in the cluster. The results suggested that *CUX1* individuals might share a certain degree of similarity, but some individuals presented heterogenous facial phenotypes.

**Fig. 1:**
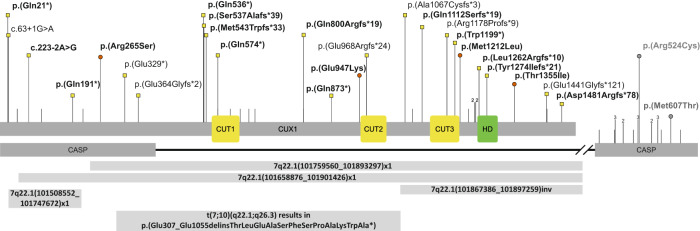
Overview of CUX1 variants. Location of missense and null variants in *CUX1* with respect to the domain structure of CUX1 (GenBank: NM_001202543.2) and CASP (GenBank: NM_001913.4). Variants reported in this cohort are labeled with the corresponding p- or c-code and are indicated by a red circle (missense) or a yellow square (null variant). Variants that affect only CASP are labeled in gray, as the relevance of those variants is uncertain. Confirmed de novo variants are indicated in bold. Lines above the protein scheme indicate null variants in gnomAD with allele count (1 is not shown). Gross deletions and structural variants are indicated as bars below the protein scheme. Abbreviations: CUT: CUT domain, HD: CUT homeobox.

We also identified two individuals with a de novo missense variant in *CUX1* that affects only the transcript encoding CASP but not CUX1 (Table S1, Fig. 1). The first individual (CASP_1:c.1820T > C, p.(Met607Thr)), had congenital glaucoma and short stature but no neurological symptoms. In contrast, a second individual (CASP_2:c.1570C > T, p.(Arg524Cys)) had severe global developmental delay, hypotonia, and seizures. It is yet unclear whether these variants are causative and whether variants in CASP are associated with another distinct disorder. Therefore, we did not include these individuals in the phenotypic description of the present cohort. In addition, we gathered information on two neonates with a de novo heterozygous null variant in *CUX1*. However, for the phenotypic characterization, we included only postnatal individuals.

### CUX1 genotypic spectrum

In 30 individuals, we identified a heterozygous *CUX1* null variant: two splice-site variants, three gross deletions, one inversion, one translocation, eight nonsense, and 15 frameshift variants. Four individuals harbor heterozygous missense variants (Fig. 1). All variants were absent in gnomAD, except the variant c.2398del. Of the 29 individuals for whom we were able to conduct a segregation analysis, 22 had de novo variants, while seven inherited the variant from a milder affected parent (Table S1). All but four truncating variants will likely result in nonsense-mediated mRNA decay (NMD). The variant c.61C > T will likely escape NMD as it creates a stop codon within the first protein-coding 100 nucleotides [28]. As the next in-frame AUG codon is 195, possibly a shortened abnormal CUX1 will be translated. The variants c.3819delG causes a frameshift that affects the CUT homeobox, truncates the C-terminal end of CUX1, and leads to escape of NMD, as confirmed by mRNA analysis (Fig. S4). The variants c.4321dup and c.4440_4447del (individuals 28 and 29, respectively) cause a frameshift predicted to result in CUX1 elongation with an abnormal C-terminal end, which possibly leads to the escape of NMD. Notably, the C-terminal region of CUX1 has a regulatory function over the transcriptional activity of the p200 protein, and Caspase-L protease can cut it during the cell cycle. This cleavage increases the transcriptional activity of the CUX1 p200 protein [29]. Hence, the variants of individuals 1, 26, 28, and 29 could lead to proteins with altered transcriptional activity compared to WT CUX1. Individual 34 had an inversion with breakpoints within intron 20 and the 3’UTR of exon 24. Although it is unclear whether this variant leads to NMD, this allele likely causes impaired CUX1 activity.

Regarding the missense variants, three of these affect highly conserved amino acid residues. However, in silico predicting programs render only slightly increased scores. Furthermore, the de novo missense variant c.4064C > T (p.(Thr1355Ile)) affects a weakly conserved residue predicted to be benign by several in silico prediction programs (Table S2).

### Analysis of Cux1 heterozygous mice

To investigate the underlying mechanisms of the *Cux1* haploinsufficiency, we characterized a previously described mouse line that carries a truncating deletion in the homeodomain of *Cux1*. Although there were no previously reported alterations in the development of these heterozygous animals [13], we observed a slight reduction of early postnatal growth (Fig. S5A). Histological analysis of brain structures revealed no differences between *Cux1*^+/−^ and WT animals (Fig. S5B).

**Fig. 2:**
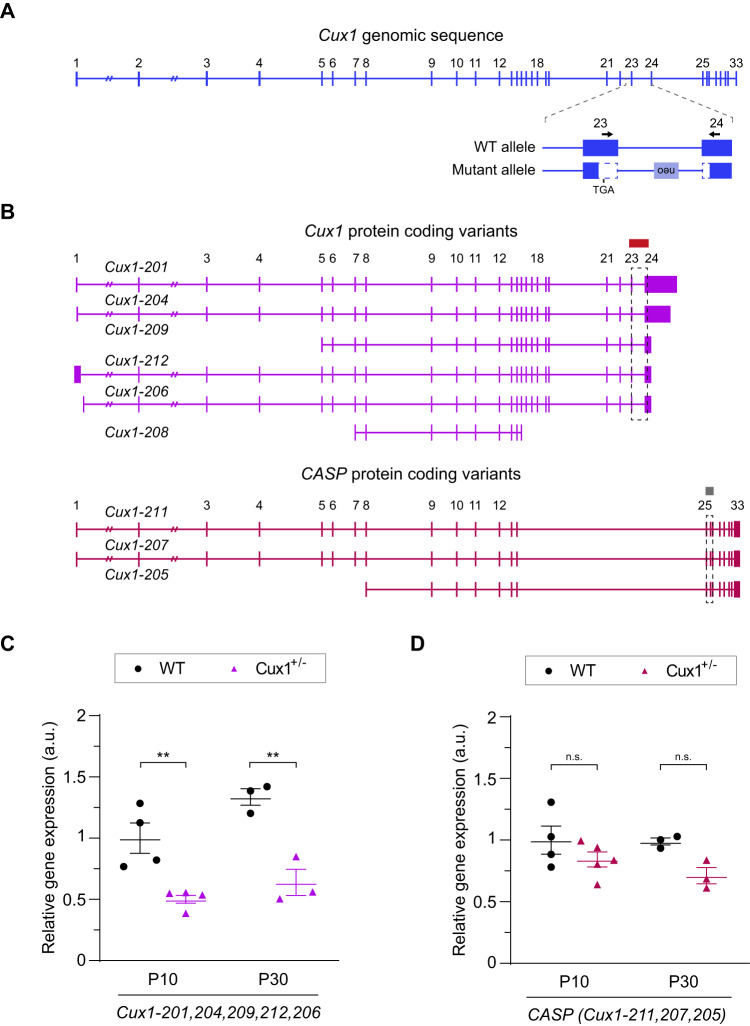
The levels of WT Cux1 transcripts are reduced in the cortex of Cux1^+/−^ mice. **A** Exon structure of *Cux1* genomic sequence and detail showing the variant deleting exons 23 and 24 in *Cux1*^+/−^. Vertical lines represent individual exons. Arrows highlight primer sequences used for the quantifications of WT transcripts by RT-qPCR. **B** Predicted transcripts coding for CUX1 and CASP. Boxes and dashed boxes highlight the regions containing the RT-qPCR amplicons used to quantify protein-coding transcripts. The *Cux1* amplicon measures all annotated CUX1 protein-coding transcripts (*Cux1-201, 204, 209, 212, 206*) except *Cux1-208*. RT-qPCR amplicon for CASP measures all annotated CASP protein-coding transcripts (*Cux1-211, 207, 205*). **C** Relative expression of *Cux1* protein-coding transcript isoforms (*Cux1-201, 204, 209, 212, 206*) as shown in (**A**) and (**B**) at P10 and P30, quantified by RT-q-PCR. Data are shown normalized to P10 WT levels. Data show mean ± SEM (*n* ≥ 3 animals per condition. Two-way ANOVA: *P*-value WT vs. Cux^+/-^ #### ≤0.0001. Post hoc with Tukey´s test: *P*-value P10 WT vs. Cux1^+/−^ ** ≤0.005, *P*-value P30 WT vs. Cux^+/−^** ≤0.005). **D** Relative gene expression of protein-coding CASP transcripts at P10 and P30. Data are shown normalized to P10 WT levels. Data show mean ± SEM (*n* ≥ 3 animals per condition. Two-way ANOVA: *P*-value WT vs. Cux^+/-^ # ≤0.05. Post hoc with Tukey´s test: *P*-value P10 WT vs. Cux^+/−^  = 0.4641 (n.s.), *P*-value P30 WT vs. Cux^+/−^  = 0.1846 (n.s.)).

We next quantified *Cux1* transcripts in WT and *Cux1*^+/−^ mouse cortices using RT-qPCR. This analysis evaluated the expression only of the WT isoforms and not the mutant transcripts (see Fig. 2A, B). As the clinical course of individuals suggests different impacts of heterozygosity at distinct developmental stages, we analyzed transcripts in the cortex of both young postnatal (P10) and mature (P30) animals. *Cux1* transcripts were reduced to half the levels of WT, both in P10 and P30 *Cux1*^+/−^cortices (Fig. 2C). As expected, as *Cux1* null alleles affect exons not present in CASP, we found no changes in the expression of CASP transcripts (Fig. 2D). This indicates that the expression of the WT allele is not upregulated to compensate for the null allele in *Cux1*^+/−^ mice.

**Fig. 3:**
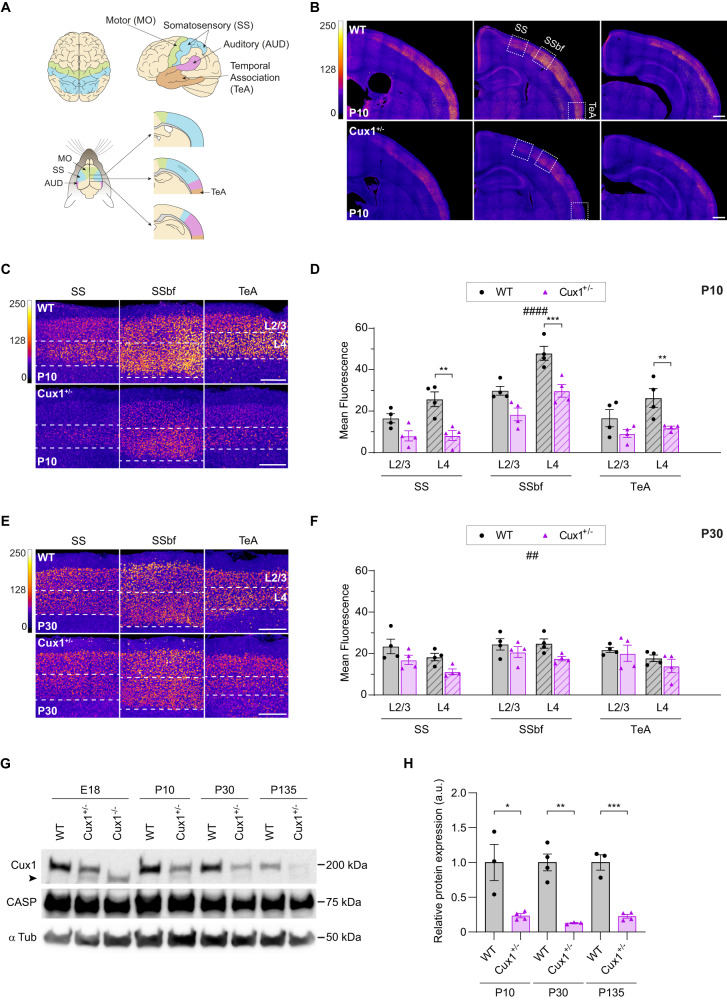
Cux1 cortical expression is reduced in heterozygous mice. **A** Comparative scheme of distinct functional areas in human (top) and mouse (bottom) brains. Top, dorsal (left), and lateral (right) views of motor (MO, green), somatosensory (SS, blue), auditory (AUD, magenta), and temporal association (TeA) cortical areas in the human brain. Bottom, dorsal (left), and medio-lateral views at several anteroposterior coordinates (right) of the functional areas in the mouse brain. **B** Intensity maps of *Cux1* expression early in development (P10) in WT and *Cux1*^+/−^ mouse brains. Images show coronal sections from more anterior (left) to more posterior (right) coordinates. Dashed boxes in the middle images highlight areas of interest (SSbf, somatosensory barrel field). Scale bar = 500 µm. **C**, **E** Magnified images of cortical upper-layer neurons (L2-4) from dashed areas of Fig. S1B at P10 (**C**) and P30 (**E**). Scale bar = 200 µm. **D**, **F** Quantification of *Cux1* expression in upper-layer neurons of SS, SSbf, and TeA areas at P10 (**D**) and P30 (**F**). Data show mean ± SEM (*n* ≥ 3 animals per condition, *n* = 2 sections per brain. P10 Two-way ANOVA: *P*-value WT vs. Cux1^+/−^^####^ ≤ 0.0001. Post hoc with Sidak´s test: *P*-value _SSL4_ WT vs. Cux1^+/−^ ** ≤ 0.01, *P*-value _SSbfL4_ WT vs. Cux^+/−^ *** ≤ 0.001, *P*-value TeAL4 WT vs. Cux^+/−^ ** ≤ 0.01. P30 Two-way ANOVA: *P*-value WT vs. Cux1^+/−^^##^ ≤ 0.01.). **G** Western blot showing cortical expression of the full-length 200 kDa CUX1 in WT, *Cux1*^+/−^, and *Cux1*^−^^/−^ (E18 only) mice at E18, P10, P30, and P135. The amount of protein was quantified and normalized to α tubulin expression (50 kDa). CASP (75 kDa), an alternatively spliced product of the *Cux1* gene, is also recognized by this antibody. The truncated mutant CUX1 is indicated by an arrowhead. **H** Relative cortical expression of the 200 kDa CUX1 at P10, P30, and P135. Data show mean ± SEM. (*n* = 3–4 cortical samples per condition. P10 unpaired *t* test: *P*-value WT vs. Cux1^+/−^ * ≤ 0.05. P30 unpaired *t* test: *P*-value WT vs. Cux1^+/−^ ** ≤ 0.01. P135 unpaired *t* test: *P*-value WT vs. Cux^+/−^ *** ≤ 0.001).

To investigate CUX1 expression in the cortex of heterozygous mice, we immunostained brain coronal sections of P10 and P30 animals and quantified CUX1 levels in several cortical areas and layers, using an antibody against the C-terminal region of CUX1 but not CASP (Fig. 3). We observed a significant reduction of CUX1 immunoreactivity in P10 *Cux1*^+/−^ mice compared to WT animals, especially in L4 neurons (Fig. 3C, D). In contrast, there was no significant difference of CUX1 immunoreactivity in P30 brains (Fig. 3E, F).

As different CUX1 isoforms show different transcriptional activity [1, 4], we analyzed protein expression using western blot. Of note, the antibody used for immunofluorescence is unfortunately unsuitable for western blot. The available antibody for immunoblotting recognizes the common N-terminal region included in p200 CUX1 and CASP but not the shorter CUX1 isoforms. This analysis demonstrated a significant reduction in the expression of p200 CUX1 in *Cux1*^+/−^ mice compared to WT at all tested ages (Fig. 3G, H). The blots also confirmed that CASP expression is unaffected in *Cux1*^*+/−*^ mice (Fig. 3G). As control of antibody specificity, we confirmed the absence of the p200 CUX1 band in lysates from E18 *Cux1*^−/−^ embryos. In both *Cux1*^*−/−*^ and *Cux1*^+/−^ cortices, we also detected low levels of the mutant truncated CUX1 reported in previous studies [13] (Fig. 3G).

Thus, western blots showed equal reductions of p200 CUX1 expression at all ages. At the same time, immunofluorescence indicated a more significant deficiency of total CUX1 during development than in adulthood. These observations suggest that the lower immunofluorescence reductions in adults are likely due to immunoreactivity from short CUX1 isoforms. As P10 and P30 *Cux1*^+/−^ mice show decreases in all transcripts, it is conceivable that post-transcriptional mechanisms balance the amount of shorter CUX1 isoforms in older animals by proteolysis of p200 CUX1.

**Fig. 4:**
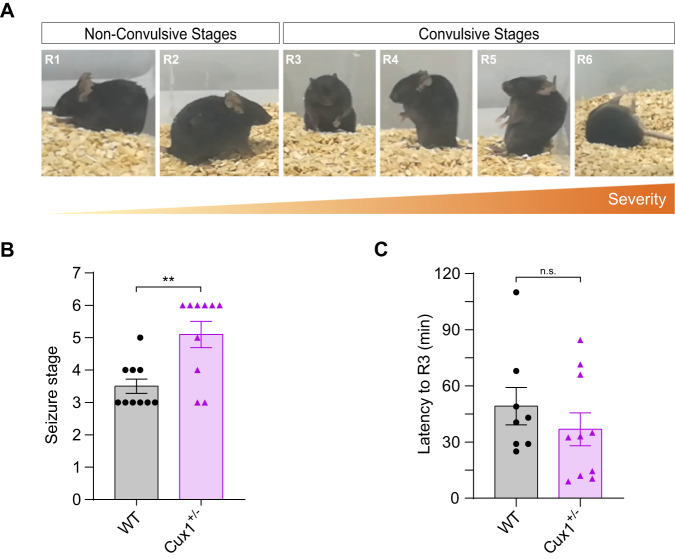
Cux1 heterozygosity results in increased seizure susceptibility in mice. **A** Identification of stages of seizure in a kainate-induced model of epilepsy. Photographs show representative behaviors from the less severe to the most, progressing from non-convulsive to convulsive seizure stages (R1-R6). **B** The maximum stage of seizure was reached in WT and *Cux1*^+/−^ mice during the first 2 h of kainate induction. Data show mean ± SEM (*n* = 10 per condition. Mann–Whitney test: *P*-value WT vs. Cux1^+/−^ ** ≤ 0.01). **C** Latency to the onset of convulsive stages (R3) in WT and *Cux1*^+/−^ mice after kainate induction. Data show mean ± SEM (*n* ≥ 8 per condition. Mann–Whitney test: *P*-value WT vs. Cux^+/−^ = 0.3478 (n.s.)).

Finally, as eight individuals in the cohort had seizures, we analyzed the susceptibility of *Cux1*^+/−^ mice to seizures upon administration of kainic acid (Fig. 4A). Only *Cux1*^+/−^ and not WT animals developed severe attacks (Fig. 4B). There was, however, no difference in latency to the intermediate epileptic stage R3 (Fig. 4C). Thus, correlating with clinical findings, *Cux1*^+/−^ mice demonstrated increased epileptic susceptibility compared to WT.

## Discussion

In the present study, we describe 34 individuals with *CUX1* variants showing haploinsufficiency and refine the phenotypic spectrum of the *CUX1*-related neurodevelopmental disorder (NDD), including 11 previously published individuals [14–16]. Disease-causing variants in *CUX1* lead to a non-specific phenotype with mild to moderate developmental delay/ID with possible developmental catch-up. 31 and 24 out of 32 assessable individuals presented with speech and motor delay, respectively. Among the individuals for whom information was available (*n* = 27), 21 exhibited a variable degree of ID, and 13 out of 31 and 8 out of 32 assessable individuals presented with hypotonia or seizures, respectively. Intrafamilial variations were observed in three out of six families (individuals 6, 7, 18, 19, 20 and 22 were comparable affected; Table S1). Generally, in these families, a null variant was transmitted from mildly affected parents to children with mild to moderate ID. Given that null variants follow a comparable pathomechanism, we also observed intrafamilial variations. In 23 families with null variants, ranged the degree of cognitive impairment from learning difficulties to moderate ID (Table 1, and Table S1). We observed additional non-neurological symptoms, such as joint laxity and short stature (Table 1). We were unable to identify any recurring specific facial dysmorphic features but the facial analysis showed a significant similarity between individuals 6, 7, 25 and 28 (Fig. S3). Although speculative, it is possible that a combination of multiple minor facial features resulted in a match of these individuals. This question can only be answered by analysing a large number of images of affected individuals using approaches such as GestaltMatcher [24]. Furthermore, our results with the mouse model demonstrate a reduction of *Cux1*/CUX1 expression in heterozygotes and support a causative link between disease-causing *CUX1* variants and epilepsy.

The present study confirms that null variants in *CUX1* are a cause for NDD. Moreover, we also provide evidence for missense variants to be causative, as we described four individuals with de novo missense variants in *CUX1* (Fig. 1). *CUX1* is intolerant to missense variation, as significantly fewer germline missense variants of *CUX1* are detected in healthy controls in gnomAD than expected (z-score for missense variants = 3.75 [22]). Remarkably, the missense variant (p.(Arg265Ser)) of individual 5 has also been reported as de novo in an individual with NDD (DDD study [30]), but we could not obtain a detailed clinical description of this individual. Structural modeling of the missense variants was not possible due to the lack of an available protein model fitting the CUX1 isoform (encoded by NM_001202543.2). CUX1 exhibits high sequence conservation with CUX2, another member of the same family of homeodomain transcription factors. Like *CUX1*, *CUX2* is highly expressed in the cerebral cortex, and both are involved in the dendritic development of L2/3 neurons [9, 31]. A recurrent de novo missense variant within the first CUT repeat of CUX2 causes *CUX2*-related epileptic encephalopathy [32]. Recent reports found other *CUX2* missense variants as genetic contributors to kainic acid-induced epilepsy [33]. Although the number of individuals with missense variants is very low, it is striking that all of them had seizures (Table 1). Moreover, our mouse studies suggested that epilepsy is part of *CUX1*-related NDD, thus supporting the notion that missense variants in *CUX1* could indeed be causative. Nevertheless, further studies are needed to clarify whether missense variants cause *CUX1*-related NDD.

*CUX1* encodes two alternative splicing proteins, CASP and CUX1. In contrast to CUX1, CASP contains no DNA binding domains and is presumed to be involved in the transport of Golgi enzymes [34]. In the present cohort, individuals 1–9 and 29–32 harbor variants that affected shared CASP and CUX1 exons, while the variants of individuals 10–28 and 33 involved only exons encoding for CUX1 (Fig. 1). Interestingly, the proportion of individuals affected by motor developmental delay was higher in those who harbored a *CUX1* variant that affected both proteins (12/13, 92%) compared to individuals with a variant that affected only CUX1 (14/20, 70%). Therefore, it is both possible that CASP contributes to the regulation of neuronal development or disruption of CASP-specific exons alters the expression of CUX1 [35]. Notewothly, there is an increased density of truncating variants in the general population (gnomAD) in the CASP-specific C-terminal region (Fig. 1). As we collected individuals with a variant that affects only CASP and observed a phenotype different from the present cohort, future studies are needed to clarify the involvement of CASP variants.

Although most individuals in the current study had mild to moderate developmental delay/ID, we also identified two neonates with a severe phenotype. Individuals F1 and F2 died shortly after birth in the context of status epilepticus and refractory respiratory disorder, respectively. In both individuals, we found a de novo frameshift variant in *CUX1* (Table S1). Based on the phenotypic-genotypic spectrum described in this study, it remains uncertain whether these de novo variants alone are causative. However, as *Cux1*^*-/-*^ mice died shortly after birth, we cannot exclude a second undetected variant in trans or a hypomorphic allele in these individuals, resulting in a complete loss of *CUX1* function.

Haploinsufficiency appears in genes that cannot fulfill their function with half the dose and whose expression cannot be homeostatically regulated by increasing transcription or reducing protein elimination [36]. On the other hand, in some cases, mutant proteins can act as dominant-negative factors in heterozygous and produce adverse phenotypes [37]. Overall, our data in *Cux1*^*+/−*^ mice supports that the deficit in CUX1 proteins is a primary cause of cognitive disabilities in affected individuals. However, we found low but detectable levels of the truncated protein in heterozygous mice (Fig. 3G). Therefore, we cannot rule out that mutant proteins contribute to abnormal phenotypes in mice and humans.

Noteworthy, the reduction in the p200 CUX1 isoform is higher than the 50% decrease expected in a heterozygous. Mechanistically, perhaps this reduction results from abnormal activation of the proteolytic cleavage of p200 described in proliferating cells [1], to compensate for the loss of shorter isoforms. On the other hand, the deficits in CUX1 expression in young *Cux1*^+/−^ L4 neurons, which upregulate CUX1 expression upon sensory experience [25, 38], and the epileptic phenotype in adults, suggest that heterozygosis compromises the upregulation of *CUX1* that takes place during activity-dependent responses. However, confirming this hypothesis requires further investigation and needs to be addressed in an independent study.

Finally, another notable observation is the minor differences in total CUX1 expression between P30 *Cux1*^+/−^ and WT mice compared to the differences in P10 animals. This observation opens possible speculations on the disease mechanism, considering the developmental catch-up observed in seven individuals. We do not have a record of the disease trajectory of these individuals, but their cognitive functions were less affected than the others without catch-up (Table S1). Although one would expect null variants in *CUX1* to be more common in the general population due to a variable expressivity or developmental catch-up, respectively, none of the variants occur in gnomAD, except for variant c.2398del (p.(Gln800Argfs*19)). To our knowledge, such a developmental catch-up is rarely seen in NDD individuals, but as genetic testing increasingly includes individuals with mild symptoms, such phenotypes may become more evident.

The limitations of this study are the unavailability of comprehensive phenotypic information for each individual, including IQ scores and clinical follow-up information.

In conclusion, we describe 34 individuals with potential causative variants in *CUX1* and delineate the underlying neurodevelopmental disorder. Most individuals had developmental delay and ID, and some of them confirmed the previously reported unusual developmental delay. Hypotonia and, to a lesser extent, seizures are part of the phenotypic spectrum. While truncating variants made up the bulk of underlying causal variants, our data suggest that rare de novo missense variants could also lead to *CUX1*-related NDD. Furthermore, our studies in mice indicate that *CUX1*-related NDD is due to insufficient production of *CUX1* transcripts and that at the protein level, neurons of mature brains partly compensate for this reduction, which could sustain the possible amelioration of IQ reduction in affected human adults.

### Supplementary information

Table S1

Supplementary Information

## Data Availability

All data concerning this work is included in the manuscript and its supplement. Genetic variants reported in this study have been submitted to ClinVar or DECIPHER and they can be accessed using the URL https://www.ncbi.nlm.nih.gov/clinvar/ (Individual ID: SCV001335372.1; SCV001431662.1; SCV002764608; SCV002764609; SCV002764610; SCV002764611; SCV002764612; SCV002764613; SCV002764614; SCV001572223.1; SCV001335373.1; SCV001335390.1; SCV002764615; SCV002764616; SCV001335391.1; SCV002764617; SCV002764618; SCV002764619; SCV001335392.1; SCV002764620; SCV002764621; SCV002764622; SCV002764623; SCV001523945.1) and https://www.deciphergenomics.org/ (Individual ID: 401777; 387322; 503181; 338131; 322029; 503182; 503183; 503185; 503186).
