# Supplementary material for: CUX1-related neurodevelopmental disorder: deep insights into phenotype-genotype spectrum and underlying pathology
Source: Eur J Hum Genet. 2023 Aug 30;31(11):1251–60. doi: 10.1038/s41431-023-01445-2 (PMC10620399; doi:10.1038/s41431-023-01445-2)
Supplement: Supplementary file 2 — Supplementary Information [file 41431_2023_1445_MOESM2_ESM.docx]

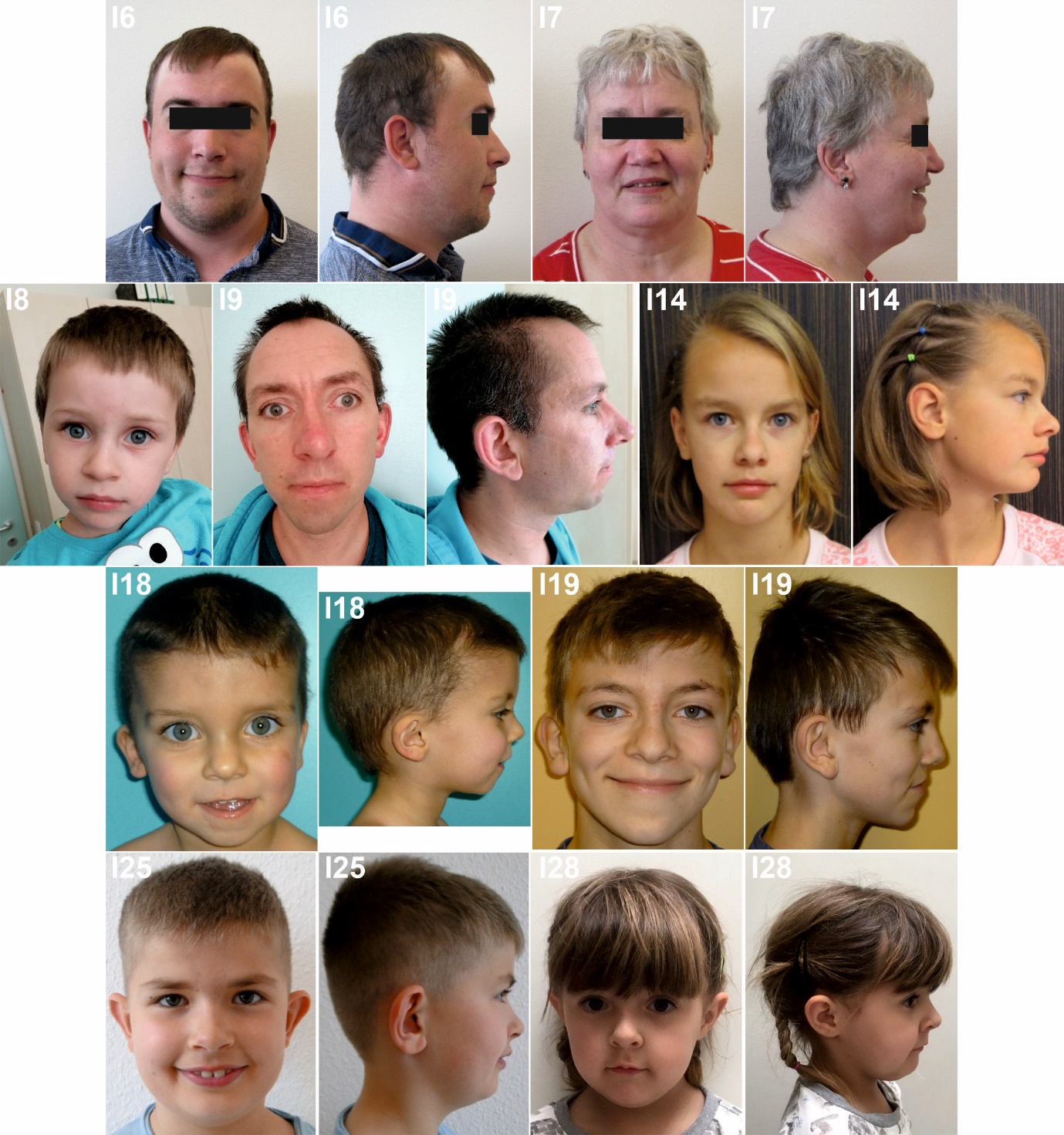


**Figure S1: Photos of individuals with *CUX1* variants.** For individual numbering, see Table 1 and Table S1


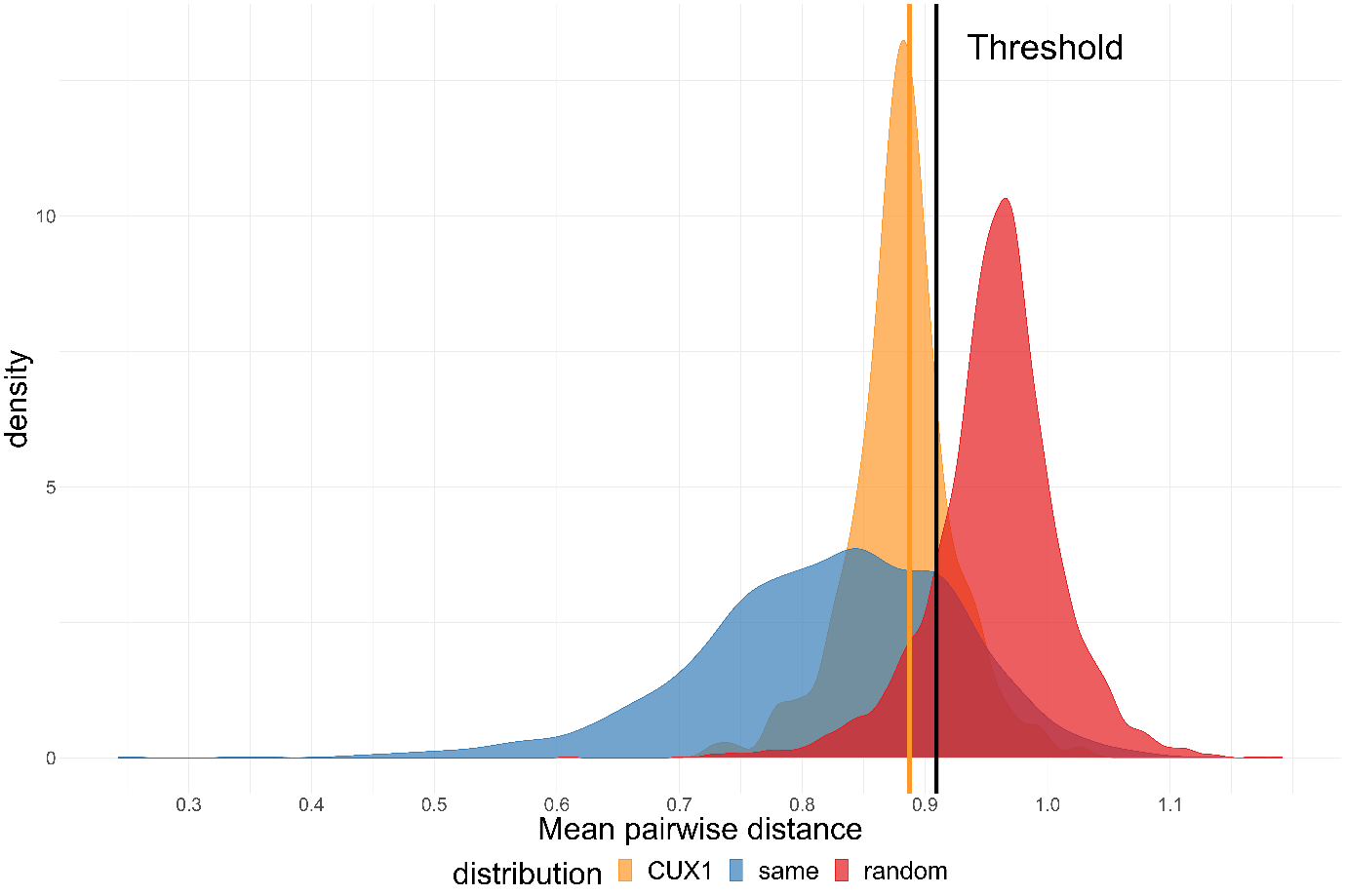


**Figure S2: The distribution of mean pairwise distance.** It shows three distributions: CUX1, the random selection from the subjects with 328 disorders, and the selection with the same disorder. The orange vertical line is the mean pairwise distance of the CUX1 cohort. The black vertical line is the threshold that classifies whether it is the same disorder or random selection. 78% of CUX1 distribution are below the threshold.


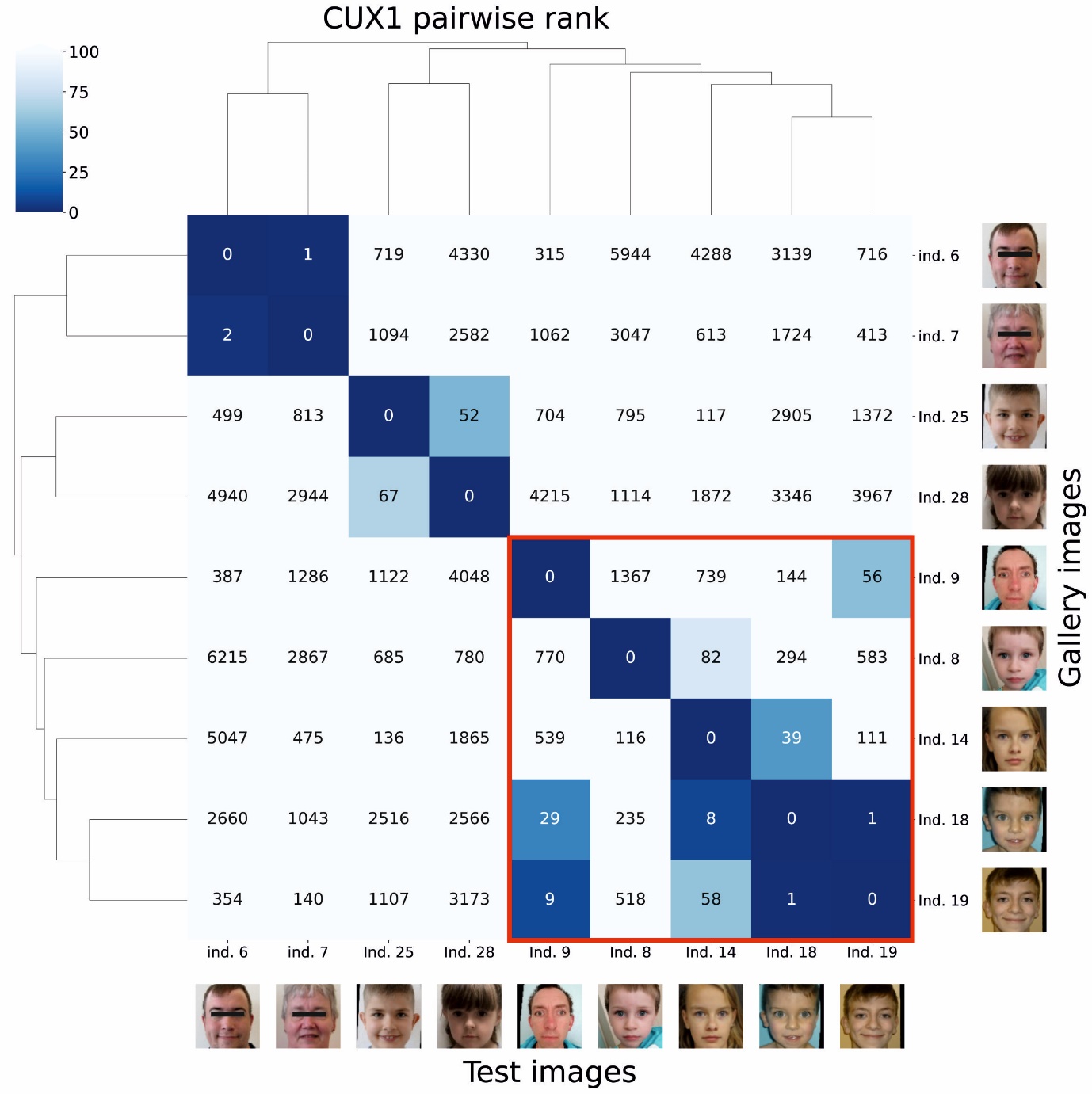


**Figure S3: The pairwise rank matrix and hierarchical clustering of nine CUX1 individuals.** Each column is the result of testing one subject in the column and listing the rank of the rest eight photos in each row. For example, by testing Ind. 9, Ind. 19 is on the 9th rank, and Ind. 18 is on the 29th rank of Ind. 9. The red box is the potential cluster of similar individuals, which indicates that this cluster shares a similar facial phenotype. Individuals 6, 7, 25, and 28 are not in the cluster, which might indicate that they show different facial phenotypes. There are three pairs of family members (ind. 6 and 7, ind. 8 and 9, ind. 18 and 19). Individuals 18 and 19 are the top-1 rank to each other might be due to coming from the same family.

***
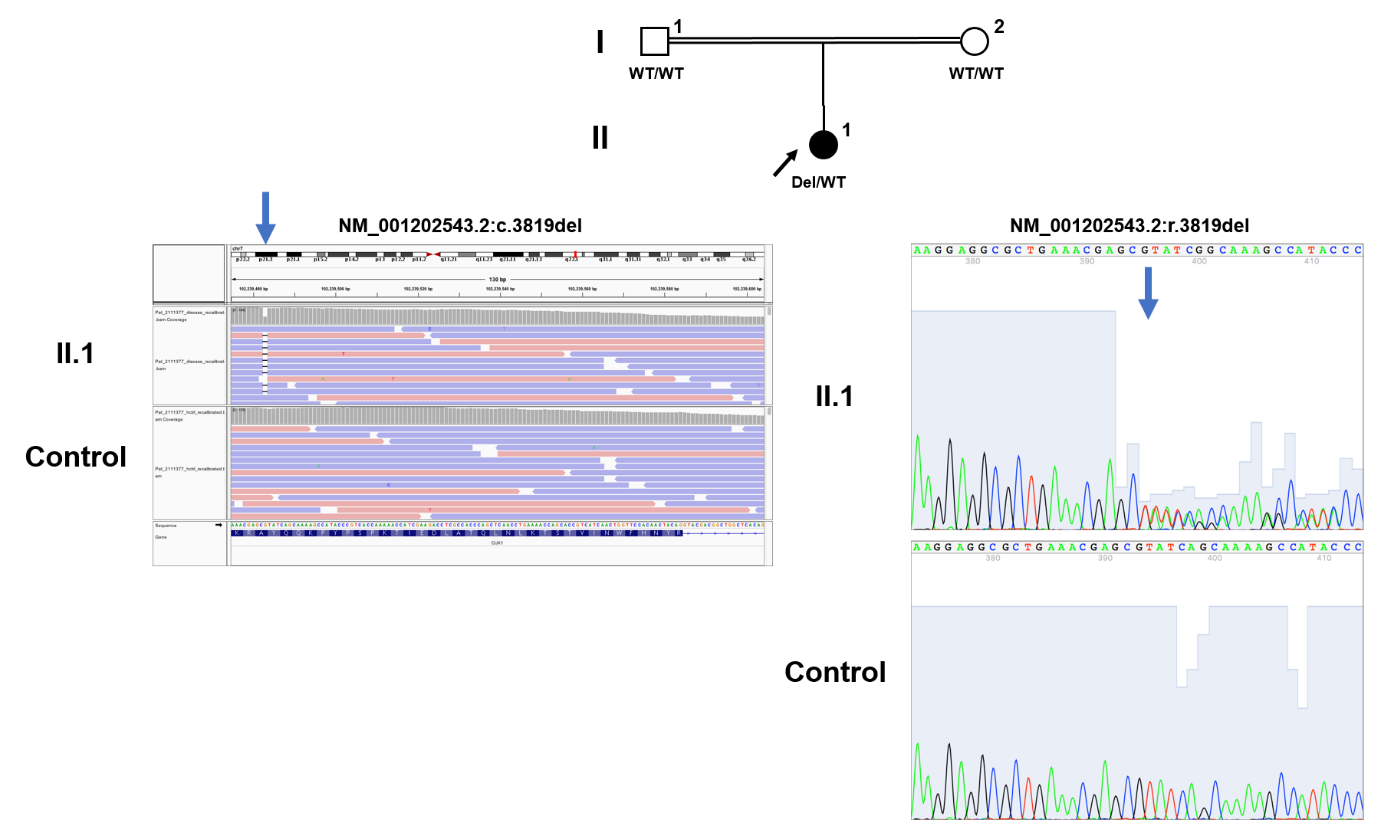
***

**Figure S4.** **The variant c.3819delG, p.(Tyr1274Ilefs*21) leads to an escape of nonsense-mediated mRNA decay.** The pedigree of individual 26 is shown. The reads of the exome sequencing (IGV) showing the heterozygous variant c.3819delG (left) and sanger sequencing confirms the heterozygous variant r.3819delG (right) in cDNA, confirming the escape of nonsense-mediated mRNA decay.


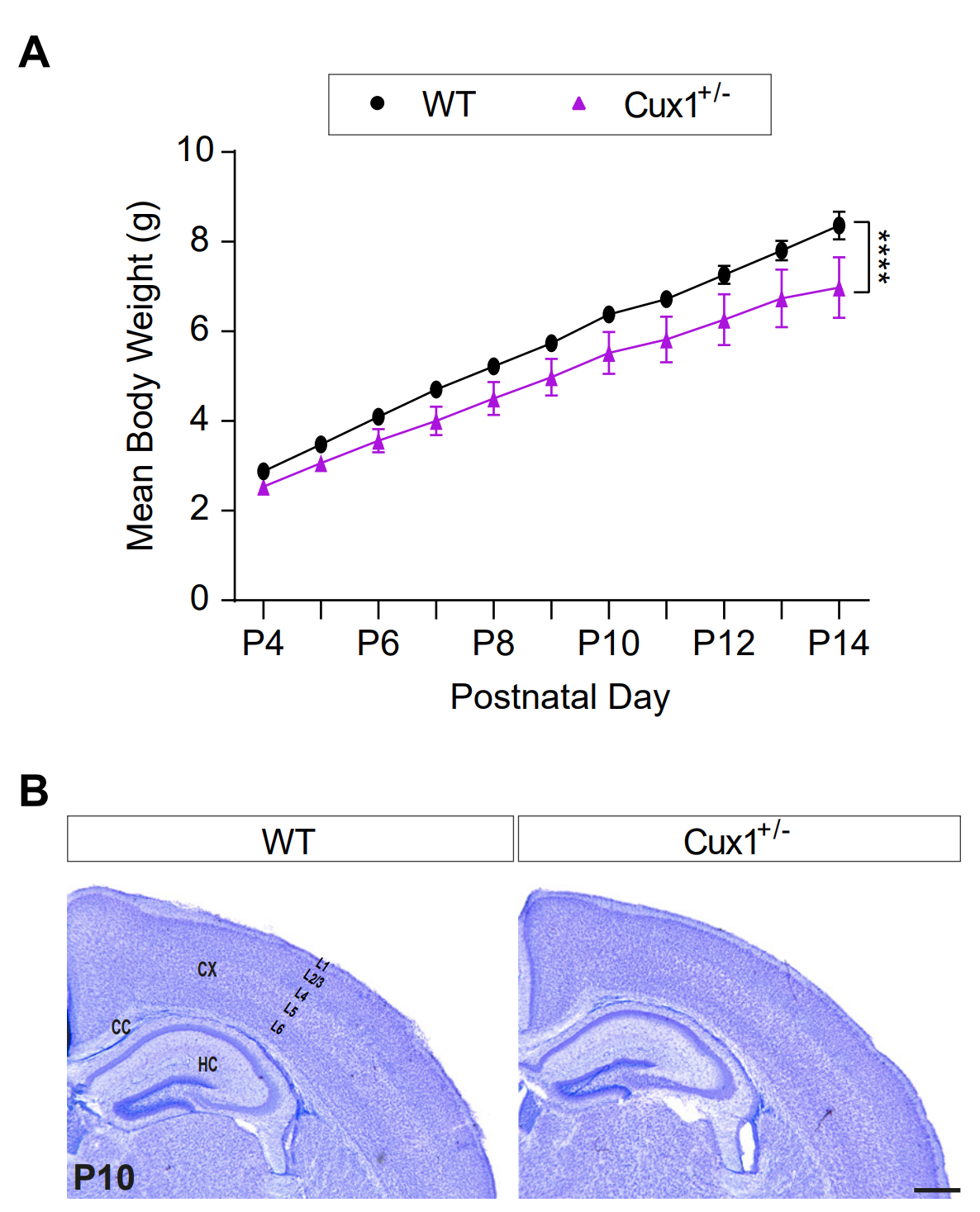


**Figure S5.** ***Cux1*^+/-^ mice exhibit no obvious structural alterations in the brain but have attenuated weight gain during development. A)** Body weight of WT and Cux1^+/-^ mice during postnatal development. Data show mean ± SEM (n = 5 animals per stage and condition. Two-way ANOVA: *P*-value _WT vs. Cux1_+/- **** ≤ 0.0001). **B)** Nissl staining of cortical sections of WT and Cux1^+/-^ mouse brains at P10. Images highlight the corpus callosum (CC), hippocampus (HC), and cortex (CX), with its distinct layers (L1-6). Scale bar = 500 µm.

Table S2: In silico scores of CUX1 missense varaints. Numbers indicated in bold that exeed the treshhold for PP3 as defined by: Pejaver et. al (1)

| **Individual** | **transcript** | **Variant description** | **Predicted protein** | **Chr7(GRCh37) position** | **Chr7(GRCh38) position** | **REVEL** | **CADD** | **MutPred2** | **Conservation** | **Absent from GnomAD** |
| --- | --- | --- | --- | --- | --- | --- | --- | --- | --- | --- |
| 5 | NM_001202543.1 | c.795G>C | p.(Arg265Ser) | g.101813764G>C | chr7:102170484 | 0.207 | 23.1 | 0.498 | moderate, frog | yes |
| 16 | NM_001202543.1 | c.2839G>A | p.(Glu947Lys) | g.101845383G>A | chr7:102202103 | 0.5 | **26** | 0.709 | moderate, frutfly | yes |
| 24 | NM_001202543.1 | c.3634A>T | p.(Met1212Leu) | g.101877499A>T | chr7:102234219 | 0.362 | **26.1** | 0.394 | high, frutfly | yes |
| 27 | NM_001202543.1 | c.4064C>T | p.(Thr1355Ile) | g.101891835C>T | chr7:102248555 | 0.094 | 17.46 | 0.156 | weak, dog | yes |

**Material and Methods**

**Facial analysis**

We performed the GestaltMatcher approach (2) on *CUX1* individuals to analyze the facial similarities among the nine individuals (Ind. 6, 7, 8, 9, 14, 18, 19, 25, and 28) who consented to the facial analysis. Each photo was first encoded into 12 512-dimensional vectors by performing model ensemble and test-time augmentation (3), and the facial phenotypic similarity between two photos was quantified by averaging 12 cosine distances between two photos. Two photos have higher facial phenotypic similarity when the distance is smaller, which means they are close in the phenotype space. We first performed the cohort-level analysis and further analyzed the similarities on the individual level.

To validate the similarities of *CUX1* individuals, we first calculated their mean pairwise distance. We further performed the random sampling 100 times. Because there are three pairs of individuals from the same family, we ensured that the family members were not sampled together to avoid the bias caused by the family trait. The distribution is shown in Figure S2 (orange). To further understand how similar the *CUX1* individuals are, we compared them to two distributions (same and random) built from the 1,555 images from different subjects with 328 different syndromes from GestaltMatcher Database (GMDB). For each of the 328 syndromes, we randomly selected a sub-cohort and calculated the mean pairwise distance 100 times to build the “same” distribution (in blue color in Figure S2). To know how the distribution of random selection is, we then randomly sampled a sub-cohort (not limit them within the same syndrome) and calculated the mean pairwise distance 100 times to build the “random” distribution (distribution in red color in Figure S2). In order to find out the threshold for distinguishing between the distribution of same and random, we performed the Receiver Operating Characteristic (ROC) analysis with five-fold cross-validation. The threshold c was chosen by the highest Youden index, resulting in c=0.909, corresponding to a sensitivity of 0.862 and a specificity of 0.792.

Ultimately, we investigated the similarities among the individuals on the individual level by performing the pairwise comparison analysis. We compared nine *CUX1* individuals to 7,459 images with 449 different disorders from GMDB by performing the leave-one-out cross-validation to simulate the real-world scenario. For example, by testing Individual 6, we put the rest eight individuals in the space with other 7,459 images and calculated the ranks of eight individuals to individual 6. With this analysis (Figure S3), we could visualize the similarity of each pair of individuals compared to the control cohort.

**Western blotting**

The somatosensory cortex was dissected in cold phosphate-buffered saline (PBS) and snap-frozen. For postnatal samples, stereotaxic injections with 1% Fast Green (Bregma coordinates: anterior-posterior axis (AP) 0, medial-lateral axis (ML) +2.5, and A.P. -2, ML +2.5 for P10; AP +0.2, ML +2.7, and AP -2.2, ML +2.7 for P30 and P135) delineated the limits of the somatosensory barrel field. Three injections of 4.6 nl were administered at depths between -0.6 and -0.1 mm, and the injection marks served to orient dissection. Tissue lysates and western blots were conducted using standard procedures (4) with antibodies against CUX1 (Proteintech, 11733-1-AP). Nonsaturated images were quantified using Fiji software (5), and relative intensity was normalized to α-tubulin expression (Sigma, T5168).

**RT-qPCR analysis**

We designed primers targeting most Cux1 protein-coding transcripts (Cux1-201, 204, 209, 212, and 206 CUX1 isoforms and Cux1-205,207, and 211 CASP isoforms) described in ENSMUSG00000029705 (6): Cux1-forward 5'-CCATCGAGGAACTTGCCACACA-3'; Cux1-reverse 5'-TTCGAGCTGAAGGTGAGTCGCT-3'; CASP-forward 5'-CAGGACCTCAGCACCATCCAGT-3'; CASP-reverse 5'-ATGGAAAGCAGGGAGTCCACCT-3'. The results were normalized using Gorasp2 (forward 5'-CTGGGAGGTTCATCTTTGGA-3'; reverse 5'-AATGTTGCATGTGGGCACTA-3').

**Confocal imaging, microscopy, and quantification**

Images were taken using a Leica DM4B (Leica) with a 2.5X objective and LAS AF v1.8 software (Leica). 50 μm optical z-sections were obtained by taking 3.5 μm serial sections with LAS AF v1.8 software (Leica) using a 512 x 512 scan format with a 20x objective. The maximum threshold was set by conditions reaching signal saturation in WT. Background noise was determined as previously reported (7,8). We manually delineated regions of interest in tilescans, and measured mean fluorescence by using Fiji (5).

**References**

1. Pejaver V, Byrne AB, Feng BJ, Pagel KA, Mooney SD, Karchin R, et al. Calibration of computational tools for missense variant pathogenicity classification and ClinGen recommendations for PP3/BP4 criteria. Am J Hum Genet. 2022 Dec 1;109(12):2163–77.

2. Hsieh TC, Bar-Haim A, Moosa S, Ehmke N, Gripp KW, Pantel JT, et al. GestaltMatcher facilitates rare disease matching using facial phenotype descriptors. Nat Genet. 2022 Mar;54(3):349–57.

3. Hustinx A, Hellmann F, Sumer O, Javanmardi B, Andre E, Krawitz P, et al. Improving Deep Facial Phenotyping for Ultra-rare Disorder Verification Using Model Ensembles. In: 2023 IEEE/CVF Winter Conference on Applications of Computer Vision (WACV) [Internet]. Waikoloa, HI, USA: IEEE; 2023 [cited 2023 Apr 26]. p. 5007–17. Available from: https://ieeexplore.ieee.org/document/10030218/

4. Cubelos B, Sebastián-Serrano A, Beccari L, Calcagnotto ME, Cisneros E, Kim S, et al. Cux1 and Cux2 regulate dendritic branching, spine morphology, and synapses of the upper layer neurons of the cortex. Neuron. 2010 May 27;66(4):523–35.

5. Schindelin J, Arganda-Carreras I, Frise E, Kaynig V, Longair M, Pietzsch T, et al. Fiji: an open-source platform for biological-image analysis. Nat Methods. 2012 Jul;9(7):676–82.

6. Cunningham F, Allen JE, Allen J, Alvarez-Jarreta J, Amode MR, Armean IM, et al. Ensembl 2022. Nucleic Acids Res. 2022 Jan 7;50(D1):D988–95.

7. Rodríguez-Tornos FM, Briz CG, Weiss LA, Sebastián-Serrano A, Ares S, Navarrete M, et al. Cux1 Enables Interhemispheric Connections of Layer II/III Neurons by Regulating Kv1-Dependent Firing. Neuron. 2016 Feb 3;89(3):494–506.

8. Briz CG, Navarrete M, Esteban JA, Nieto M. In Utero Electroporation Approaches to Study the Excitability of Neuronal Subpopulations and Single-cell Connectivity. J Vis Exp JoVE. 2017 Feb 15;(120).
